# Mobility Models Based on Forward Current-Voltage Characteristics of P-type Pseudo-Vertical Diamond Schottky Barrier Diodes

**DOI:** 10.3390/mi11060598

**Published:** 2020-06-18

**Authors:** Min-Woo Ha, Ogyun Seok, Hojun Lee, Hyun Ho Lee

**Affiliations:** 1 Department of Electrical Engineering, Myongji University, 116 Myongji-ro, Cheoin-gu, Yongin, Gyeonggi 17058, Korea; isobar@mju.ac.kr (M.-W.H.); 80180234@mju.ac.kr (H.L.); 2 Korea Electrotechnology Research Institute, Changwon, Gyeongnam 51543, Korea; ogseok@keri.re.kr; 3 Department of Chemical Engineering, Myongji University, 116 Myongji-ro, Cheoin-gu, Yongin, Gyeonggi 17058, Korea

**Keywords:** diamond, Schottky barrier diode, power device, hole mobility, forward current

## Abstract

Compared with silicon and silicon carbide, diamond has superior material parameters and is therefore suitable for power switching devices. Numerical simulation is important for predicting the electric characteristics of diamond devices before fabrication. Here, we present numerical simulations of p-type diamond pseudo-vertical Schottky barrier diodes using various mobility models. The constant mobility model, based on the parameter *μ_const_*, fixed the hole mobility absolutely. The analytic mobility model resulted in temperature- and doping concentration-dependent mobility. An improved model, the Lombard concentration, voltage, and temperature (CVT) mobility model, considered electric field-dependent mobility in addition to temperature and doping concentration. The forward voltage drop at 100 A/cm^2^ using the analytic and Lombard CVT mobility models was 2.86 and 5.17 V at 300 K, respectively. Finally, we used an empirical mobility model based on experimental results from the literature. We also compared the forward voltage drop and breakdown voltage of the devices, according to variations in p- drift layer thickness and cathode length. The device successfully achieved a low specific on-resistance of 6.8 mΩ∙cm^2^, a high breakdown voltage of 1190 V, and a high figure-of-merit of 210 MW/cm^2^.

## 1. Introduction

Diamond is a promising semiconductor for power switches due to its wide band gap, high critical field, and high thermal conductivity [1,2,3]. Diamond has a wide band gap of 5.5 eV, which corresponds to the boundary between semiconductors and insulators. Diamond’s high breakdown field (> 10 MV/cm) enables a thin drift layer and low on-resistance in power devices. Its thermal conductivity is 22 W/cm/K, which is the highest of any material on earth. Diamond could replace Si and SiC in conventional power devices, especially for high-temperature and high-power applications [4,5,6,7,8,9,10,11].

The ionization energies of phosphorous for n-type diamond and boron for p-type diamond are 570 and 370 meV at room temperature, respectively [12]. When boron concentration increases, the ionization energy decreases due to the change in carrier transport properties from band transport to hopping conduction [12,13]. Operating diamond power devices at elevated temperatures can also increase the number of ionized carriers [14]. Technologies for growing and processing diamond are still in development. A pseudo-vertical diode enabled a local vertical current flow, which was useful considering the level of technology at that time. A pseudo-vertical diamond diode that enabled local vertical current flow was demonstrated; the diode had a 14 μm-thick p-layer, was fabricated using an etching process, and exhibited a high breakdown voltage of 1.6 kV and low leakage current of 10^−7^–10^−6^ A/cm^2^ [15]. The electrical characteristics of this device were recorded using a field plate on an Al_2_O_3_ dielectric layer [16]. A diamond device with high dislocation density was found to exhibit a high leakage current, as shown using a defect visualization technique [17], while a diamond PIN diode with a 8.5-μm-thick intrinsic layer on IIa-type (100) bulk substrate was found to have an experimental breakdown voltage of 1040 V [18].

Simulations and numerical models can be used to forecast the electrical characteristics of devices before fabrication. Such experimental approaches are important; however, a simulation technology for diamond still needs to be developed. Basic parameters and models for diamond devices have been discussed [19]; a simulation of junction termination structures in diamond Schottky barrier diodes (SBDs) was discussed in [20,21]; simulation models for diamond bipolar devices were analyzed and discussed in detail in [22]; and, three-dimensional simulations of depleted Schottky PIN diamond diodes were performed in [23]. A quantum simulation of a nitrogen-terminated diamond (111) surface has also been reported [24], and a diamond Schottky PIN and SBD have been simulated with breakdown voltages from 70 to 5000 V [4]. We previously reported the simulated breakdown voltage of p-type diamond pseudo-vertical SBDs with modified impact ionization coefficients [25].

In this paper, we focus on the forward current–voltage (I–V) characteristics of p-type pseudo-vertical diamond SBDs using mobility models. Identical devices were simulated using constant, analytic [26,27], Lombardi CVT (concentration, voltage, and temperature) [28], and empirical mobility models [22]; the empirical mobility model was based on Volpe’s experiment [29]. The mobility models yielded different forward I–V characteristics due to differences in the degree of dependence on temperature, doping concentration, and electric field. We chose the Atlas numerical simulation tool within the Silvaco software [30] to investigate the forward voltage drop (*V_f_*), specific on-resistance (*R_on,sp_*), and breakdown voltage of p-type pseudo-vertical diamond SBDs.

## 2. Numerical Methods

**Figure 1:**
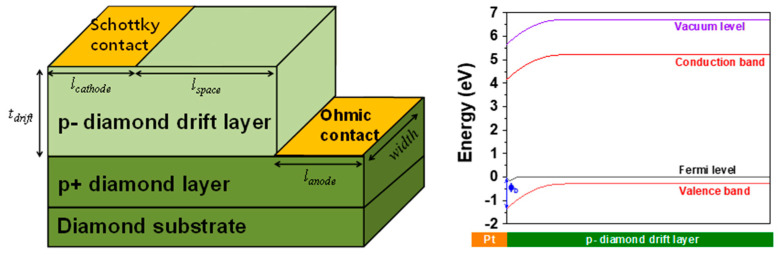
Cross-sectional view of the p-type diamond pseudo-vertical Schottky barrier diode (SBD) for forward current–voltage (I–V) and breakdown voltage. Simulated energy band at the Schottky contact/p- diamond drift layer at 300 K is also shown.

The device structure was designed in a sequence of defining mesh, region, electrode, doping, and contact. We defined various material parameters of the p-type diamond, choice models with physical equations, and selected numerical methods to solve the equations, and finally swept the voltage on the electrodes. Values of 5.5 eV, 1.5 eV, 5.7 × 10^−9^ s, and 5 × 10^19^ cm^−3^ were used for the energy band gap, affinity, permittivity, Shockley-Read-Hall hole lifetime, and room temperature density of states in the valence band, respectively [31]. Figure 1 shows the cross-sectional view and simulated energy band at the Schottky contact of the p-type diamond pseudo-vertical SBD. The voltage was not applied in the energy band at 300 K. The hole current flows vertically from the Schottky contact (cathode) to the interface between the p- drift layer and the p+ layer; then, it flows laterally through the interface to the Ohmic contact (anode). The Schottky contact was platinum, with a metal work function (*ϕ_m_*) of 5.65 eV. The Schottky barrier height (*ϕ_b_*) was 1.35 eV from the Mott equation and simulation, respectively. The doping concentration of the p- drift layer (*N_a_*) was 10^15^ cm^−3^ for the high breakdown voltage. The *N_a_* increased with increasing temperature, but this was not taken into account to focus on the mobility study with temperature. It is necessary to consider the limitation of the assumption. The thickness of the p- drift layer (*t_drift_*) was 4.6 μm. The lateral length of the Schottky (*l_cathode_*) and Ohmic contacts (*l_anode_*) was 2 and 2 μm, respectively, and the spacing between Schottky and Ohmic contacts (*l_space_*) was 16 μm.

The saturation velocity of holes was fixed at 2.7 × 10^7^ cm/s so that the forward current was determined by the hole mobility. The hole mobility in the p-type low-doped diamond was controlled by ionized impurity, acoustic phonon, and optical phonon scattering [32]. We considered four mobility models related to scattering, namely constant, analytic, Lombardi CVT, and empirical mobility models. We defined *V_f_* at a current density of 100 A/cm^2^. The current density was defined as current per width divided by the sum of *l_cathode_*, *l_space_*, and *l_anode_*, and *R_on,sp_* was extracted from the reciprocal of the I–V slope between the built-in potential and *V_f_*.

## 3. Simulation Results and Discussion

### 3.1. Constant Mobility Model

The doping concentration of the p- drift layer and the saturation velocity of the holes were fixed, so that the hole mobility and forward current were affected by scattering. First, we investigated the device using the constant mobility model, which considers the mobility to be dependent only on acoustic phonon scattering due to temperature. We used Equation (1) for the constant mobility model. Here, *μ_p_*, *T*, and *μ_const_* are the hole mobility, lattice temperature, and constant mobility at 300 K, respectively. Equation (1) does not take into account any change in mobility due to the doping concentration or electric field.

(1) $$\mu_{p}\left(T\right)=\mu_{const}{\left(\frac{T}{300}\right)}^{-1.5}$$

**Figure 2:**
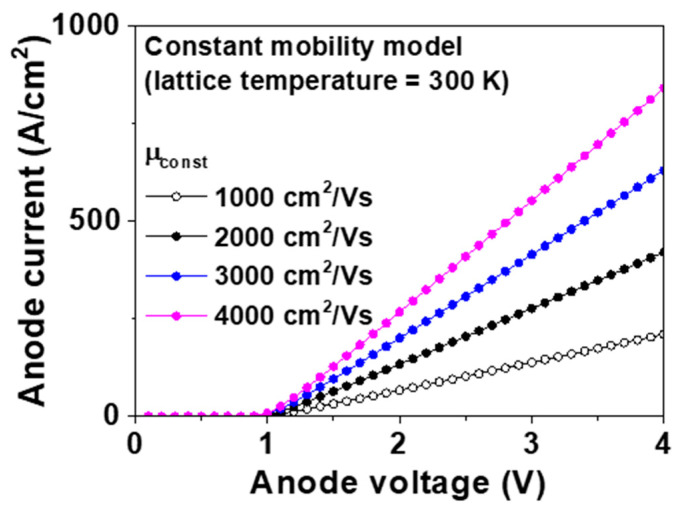
Forward current–voltage (I–V) characteristics of the p-type diamond pseudo-vertical Schottky barrier diode (SBD) simulated using the constant mobility model at 300 K. The values of *μ_const_* were 1000, 2000, 3000, and 4000 cm^2^/Vs, respectively. The metal work function, hole saturation velocity, and lattice temperature were 5.65 eV, 2.7 × 10^7^ cm/s, and 300 K, respectively.

Figure 2 shows the forward I–V characteristics of the p-type diamond pseudo-vertical SBD simulated using the constant mobility model with various *μ_const_*. The extracted *V_f_* and *R_on,sp_* is shown in Table 1. The anode voltage (*V_a_*) was swept from −10 to 10 V at 300 K. The Schottky barrier height of the p-type SBD was defined by *E_g_* − (*ϕ_m_* − *χ*). Finally, the built-in potential was calculated as the difference between the Schottky barrier height and *E_F_* − *E_v_*, where *E_F_* and *E_v_* were derived from the Fermi energy level and edge of the valence band, respectively. The built-in potential was 1.1 V at 300 K. When *μ_const_* was 1000, 2000, 3000, and 4000 cm^2^/Vs, *V_f_* was 2.47, 1.76, 1.52, and 1.40 V at 300 K, respectively. These values can be converted to values of *R_on,sp_* as 14.1, 7.0, 4.6, and 3.4 mΩ∙cm^2^ at *μ_const_* of 1000, 2000, 3000, and 4000 cm^2^/Vs, respectively. The typical mobility at the 10^15^ cm^−3^-doped p-drift layer was 1990 cm^2^/Vs from the empirical mobility model [29]. The value of *μ_const_* is important for the constant mobility model.

**Figure 3:**
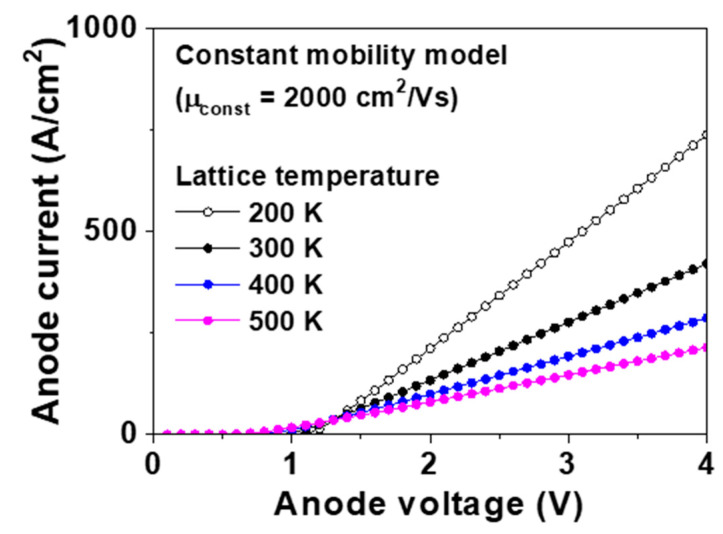
Forward current–voltage (I–V) characteristics of the p-type diamond pseudo-vertical Schottky barrier diode (SBD) simulated using the constant mobility model at 200, 300, 400, and 500 K. The metal work function, hole saturation velocity, and *μ_const_* were 5.65 eV, 2.7 × 10^7^ cm/s, and 2000 cm^2^/Vs, respectively.

For a value of *μ_const_* of 2000 cm^2^/Vs, we simulated the forward I–V characteristics at 200, 300, 400, and 500 K. Figure 3 shows the simulated forward I–V characteristics of the p-type diamond pseudo-vertical SBD with a *μ_const_* value of 2000 cm^2^/Vs at 200, 300, 400, and 500 K, respectively. The extracted built-in potential, hole mobility, *V_f_* and *R_on,sp_* are shown in Table 2. When the lattice temperature was 200, 300, 400, and 500 K and *μ_const_* was 2000 cm^2^/Vs, the values of *V_f_* were 1.57, 1.76, 2.01, and 2.30 V, respectively, and the values of *R_on,sp_* were 3.8, 7.0, 10.9, and 14.8 mΩ∙cm^2^, respectively. Low temperature increased the Schottky barrier height. However, the decrease in temperature also increased the hole mobility and forward current due to the reduced acoustic phonon scattering. Hole mobilities of 3670, 2000, 1300, and 930 cm^2^/Vs were measured at 200, 300, 400, and 500 K, respectively, with *μ_const_* set at 2000 cm^2^/Vs. The constant mobility model has limitations because it only considers acoustic phonon scattering. In addition, *N_a_* and *V_a_* do not change the hole mobility in this model.

### 3.2. Analytic Mobility Model

The constant mobility model does not take the doping concentration or electric field into account. The hole mobility using the constant mobility model was identical under different doping concentrations. The analytic mobility model can calculate the doping concentration- and temperature-dependent mobility [26,27], and considers both acoustic phonon and ionized impurity scattering. Equation (2), below, was used for this model. The parameters of *μ_min_*, *m_1_*, *C_0_*, *γ_2_*, and *n_min_* for diamond were obtained from the literature [33], and set at 55 cm^2^/Vs, −2, 1.12 × 10^14^ cm^−3^, 0.589, and 2.5, respectively. The values of *μ_max_* and *n_max_* were 4399 cm^2^/Vs and −3.4 at lattice temperatures between 343 and 600 K, and 3433 cm^2^/Vs and −1.55 at lattice temperatures below 343 K, respectively. Figure S1 in the Supplementary Material shows the forward I–V characteristics of the device using the analytic mobility model at 200, 300, 400, and 500 K. The extracted built-in potential, hole mobility, *V_f_* and *R_on,sp_* at various temperatures of the device using analytic mobility model are shown in Table 2. The values of *V_f_* were 3.16, 2.86, 2.69, and 2.57 V at 200, 300, 400, and 500K, respectively. These values can be used to calculate values of *R_on,sp_* of 19.3, 17.5, 17.4, and 17.5 mΩ∙cm^2^ at 200, 300, 400, and 500 K, respectively. The built-in potential decreased with decreasing lattice temperature. The extracted hole mobility of the p- drift layer at 200 K was 720 cm^2^/Vs which was less than those at 300, 400, or 500 K, respectively, due to the ionized impurity scattering. In addition, the hole mobility in the analytic mobility model did not change with changes in the electric field.

(2) $$\mu_{p}\left(N_{a},T\right)=\mu_{min}{\left(\frac{T}{300}\right)}^{n_{min}}+\frac{\mu_{max}{\left(\frac{T}{300}\right)}^{n_{max}}-\;\mu_{min}{\left(\frac{T}{300}\right)}^{n_{min}}}{1+{\left(\frac{T}{300}\right)}^{m_{1}}{\left(\frac{N_{a}}{C_{0}}\right)}^{\gamma_{2}}}$$

### 3.3. Lombardi CVT Mobility Model

When *V_a_* is applied, an electric field is distributed inside the p- drift layer. This distribution can vary the mobility in the p- drift layer. However, the analytic mobility model does not allow mobility variation in layers with the same doping concentrations. In contrast, the Lombardi CVT mobility model can account for such variations; for example, this model can take account of the mobility degradation in an inversion layer of metal-oxide-semiconductor field-effect transistors. The Lombardi CVT mobility model considers the perpendicular electric field, doping concentration, and temperature-dependent mobility [28]. Scattering with surface acoustic phonons, surface roughness, and optical intervalley phonons, denoted by *μ_ac_*, *μ_sr_* and *μ_b,_* respectively, all limit the mobility. Important parameters for Lombardi CVT mobility are *T*, *N_a_*, and the perpendicular electric field. The hole mobility was determined using Matthiessen’s rule:

(3) $$\frac{1}{\mu_{p}}=\frac{1}{\mu_{ac}}+\frac{1}{\mu_{sr}}+\frac{1}{\mu_{b}}$$

**Figure 4:**
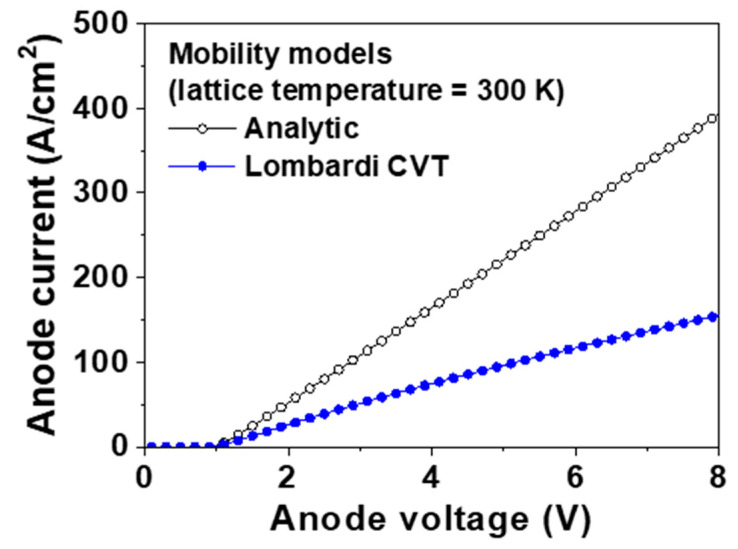
Forward current–voltage (I–V) characteristics of the p-type diamond pseudo-vertical Schottky barrier diode (SBD) simulated using the analytic and Lombardi mobility models, respectively. The metal work function, hole saturation velocity, and lattice temperature were 5.65 eV, 2.7 × 10^7^ cm/s, and 300 K, respectively.

Figure 4 shows the forward I–V characteristics of the p-type diamond pseudo-vertical SBD simulated using the analytic and Lombard CVT mobility models, respectively. The values of *V_f_* using the analytic and Lombard CVT mobility models were 2.86 and 5.17 V at 300 K, respectively. The values of *R_on,sp_* from the analytic and Lombard CVT mobility models were 17.5 and 40.8 mΩ∙cm^2^ at 300 K, respectively. When the value of *V_a_* increased up to 8 V, the forward currents with the analytic and Lombard CVT mobility models were 394 and 155 A/cm^2^, respectively. When *V_a_* increased, the forward current using the Lombardi CVT mobility model decreased compared with that using the analytic mobility model, because the Lombardi CVT mobility model considers the decrease in mobility caused by the high electric field (in particular, the electric field concentrated on the right edge of the Schottky contact that resulted in the low mobility).

**Figure 5:**
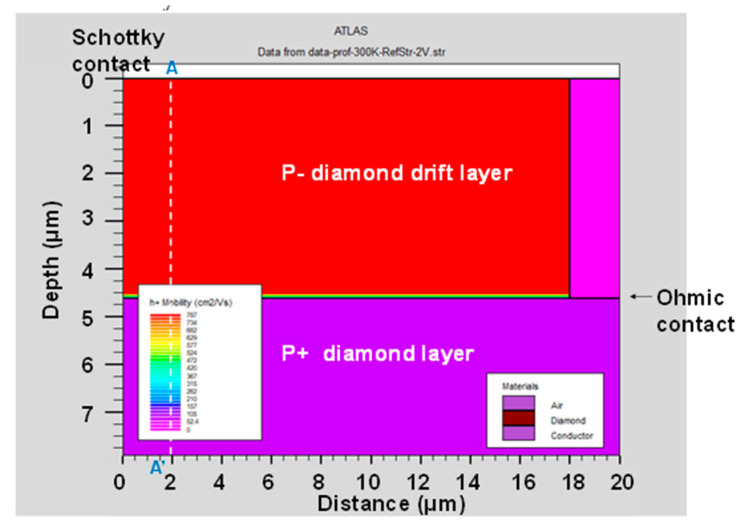
Hole mobility of the p-type diamond pseudo-vertical Schottky barrier diode (SBD) simulated using the analytic mobility model. The anode voltage (*V_a_*) was 2 V at 300 K.

**Figure 6:**
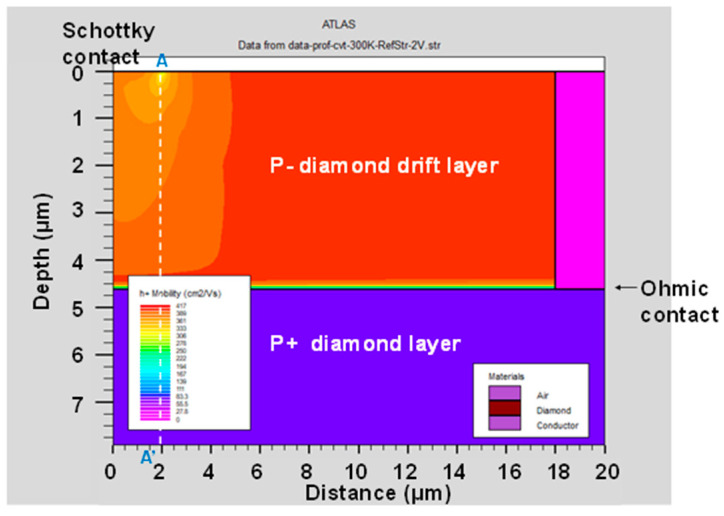
Hole mobility of the p-type diamond pseudo-vertical Schottky barrier diode (SBD) simulated using the Lombardi CVT mobility model. The anode voltage (*V_a_*) was 2 V at 300 K.

**Figure 7:**
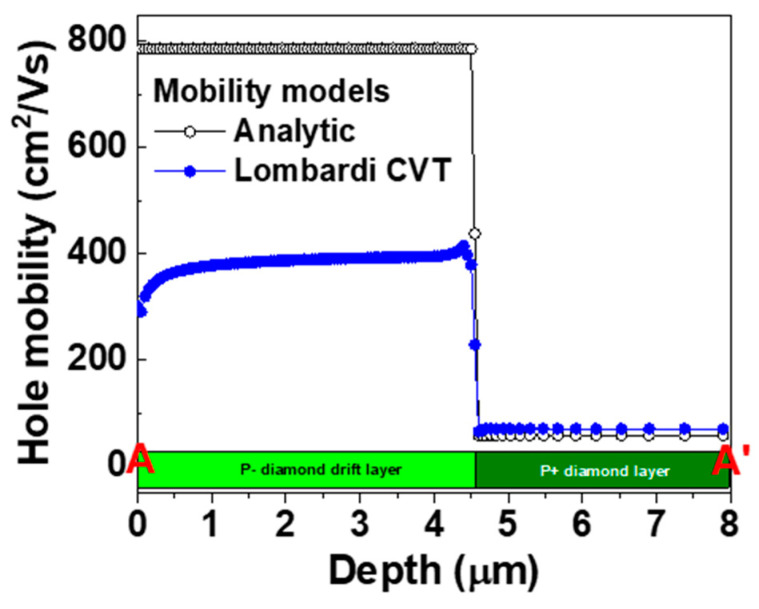
Hole mobility across the A-A’ section line of the p-type diamond pseudo-vertical Schottky barrier diode (SBD) simulated using the analytic and Lombardi CVT mobility models. The metal work function, hole saturation velocity, lattice temperature, and anode voltage (*V_a_*) were 5.65 eV, 2.7 × 10^7^ cm/s, 300 K, and 2 V, respectively.

Figure 5 and Figure 6 show the hole mobility of the p-type diamond pseudo-vertical SBD simulated using the analytic and Lombard CVT mobility models at a *V_a_* of 2 V, respectively. The analytic mobility fixes the hole mobility even if the potential or electric field is varied. However, the Lombardi CVT mobility model changes the hole mobility in response to the two-dimensional distribution of the potential and electric field. We also extracted the hole mobility across section A-A’ from Figure 5 and Figure 6, as shown in Figure 7. The hole mobility with the analytic model did not change due to the electric field. The scattering of the Lombardi CVT mobility model reduced the hole mobility. In addition, the electric field was concentrated at the edge of the Schottky contact, and the hole mobility with the Lombardi CVT mobility model decreased in this spot.

Figure S2 in the Supplementary Material shows the two-dimensional electric field of the device obtained using the Lombard CVT mobility model. The values of *V_a_* and lattice temperature were 2 V and 300 K respectively. The electric field led to a decrease in both *μ_ac_* and *μ_sr_* in the Lombardi CVT mobility model. We also simulated the forward I–V characteristics using the Lombardi CVT mobility model at 200, 300, 400, and 500 K, as shown in Figure S3 of the Supplementary Material. The extracted *V_f_* and *R_on,sp_* at various temperatures of the device using Lombardi CVT mobility model are shown in Table 2. The values of *V_f_* at 200, 300, 400, and 500 K were 2.84, 5.17, 9.11, and 15.49 V, respectively. The values of *R_on,sp_* at 200, 300, 400, and 500 K were 17.2, 40.8, 82.0, and 145.8 mΩ∙cm^2^, respectively. The decrease in lattice temperature suppressed the scattering of *μ_ac_* and *μ_b_*. The Lombardi CVT mobility model is suitable for numerical simulation of diamond devices under high electric field, because it considers the mobility reduction due to the electric field.

### 3.4. Empirical Mobility Model

**Figure 8:**
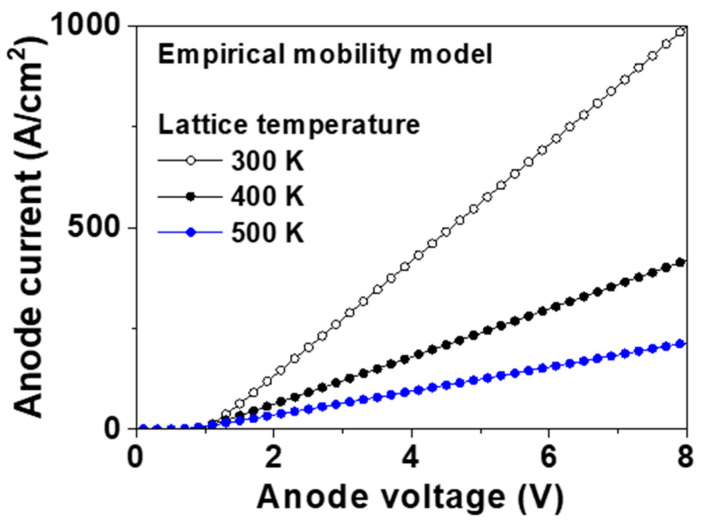
Forward current–voltage (I–V) of the p-type diamond pseudo-vertical Schottky barrier diode (SBD) simulated using the empirical mobility model at 300, 400, and 500 K. The metal work function, hole saturation velocity, hole mobility at 300 K, and *β* were 5.65 eV, 2.7 × 10^7^ cm/s, 1990 cm^2^/Vs, and 3.092, respectively.

We also investigated the hole mobility based on the experimental results from Volpe et al. [22,29]. They reported that ionized impurity scattering was the dominant scattering mechanism, and that the room temperature mobility was limited by crystalline defects. They proposed the mobility equation given below, which is based on experimental results:

(4) $$\mu_{p}\left(T,{\;N}_{a}\right)=\mu \left(300K,\;N_{a}\right){\left(\frac{T}{300}\right)}^{-\beta \left(N_{a}\right)}$$

where $\mu_{p}\left(300K,N_{a}\right)$, $\beta \left(N_{a}\right)$, *μ_max_*, *μ_min_*, *N_μ_*, γ*_μ_*, *β_max_*, *β_min_*, *N_β_*, and γ*_β_* were $\mu_{min}+\frac{\mu_{max}-\mu_{min}}{1+{\left(\frac{N_{a}}{N_{\mu }}\right)}^{\gamma_{\mu }}}$, $\beta_{min}+\frac{\beta_{max}-\beta_{min}}{1+{\left(\frac{N_{a}}{N_{\beta }}\right)}^{\gamma_{\beta }}}$, 2016 cm^2^/Vs, 0 cm^2^/Vs, 3.25 × 10^17^ cm^−3^, 0.73, 3.11, 0, 4.1 × 10^18^ cm^−3^, and 0.617, respectively, for our devices. The hole mobility at 300 K and *β(N_a_)* obtained from the empirical model were 1990 cm^2^/Vs and 3.092, respectively, for our device structure. We inputted the parameters and simulated the devices. Figure 8 shows the forward I–V characteristics of the p-type pseudo-vertical diamond SBD obtained using the empirical mobility model at 300, 400, and 500 K, respectively. The values of *V_f_* at 100 A/cm^2^ were 1.77, 2.65, and 4.20 V at 300, 400, and 500 K, respectively. These values can be converted into *R_on,sp_* values of 6.8, 16.8, and 33.7 mΩ∙cm^2^ at 300, 400, and 500 K, respectively. The hole mobilities calculated from the equation at 300, 400, and 500 K were 1990, 820, and 410 cm^2^/Vs, respectively, identical to the values extracted from the numerical simulation.

The built-in potential, hole mobility, *V_f_* and *R_on,sp_* using constant, analytic, Lombardi CVT, and empirical mobility models are compared in Table 2. The hole mobility using Lombardi CVT mobility model was not added in Table 2 because the electric field influenced the hole mobility. It is noted that the hole mobility model determined the *V_f_* and *R_on,sp_*. The scattering-related forward current using Lombardi CVT model was also adjusted by a scattering factor. We defined the scattering factor of 1 in this work. When the device operates at the high field, the Lombardi CVT mobility model is appropriate because it considers the field-dependent mobility degradation. However, the safe operating area should be limited by the maximum junction temperature, so the diamond power devices should be used at the low on-current density. The empirical mobility model is suitable, compared with the constant and analytic mobility models because it is based on experimental results. The simulated *R_on,sp_* compared with the experimental result from the literature. The simulated *R_on,sp_* of 6.8 mΩ∙cm^2^ using the empirical mobility model was less than the experimental one of 18.5 mΩ∙cm^2^ [15]. This difference was caused by the epitaxy quality and fabrication process. The diamond technology has not been optimized unlike conventional silicon, and various material and fabrication methods exist. The trap level, trap density, Fermi-level pinning, and Ohmic contact resistance were not considered in this work because the epitaxy growth and fabrication process of the diamond semiconductors are still developing. This result based on the hole mobility can help the numerical studies for the diamond devices.

### 3.5. Breakdown Voltage

We investigated the device geometry using the empirical mobility model. If one parameter was changed, the other parameters were fixed for comparison. We also simulated the breakdown voltage of the devices using Selberherr’s impact ionization model [34]. The impact ionization coefficients, *α_n_* and *α_p_*, for electrons and holes, respectively, were calculated using the following equations [35,36]:

(5) $$\alpha_{n}=A_{n}exp\left[-{\left(\frac{B_{n}}{E}\right)}^{C_{n}}\right]$$

(6) $$\alpha_{p}=A_{p}exp\left[-{\left(\frac{B_{p}}{E}\right)}^{C_{p}}\right]$$

where *A_n_*, *A_p_*, *B_n_*, *B_p_*, *C_n_*, and *C_p_* are fitting parameters and *E* is the electric field in the direction of the current. We modified the fitting parameters so that the breakdown field of the parallel-plane Schottky contact/p- drift layer of 10 MV/cm was satisfied [25].

**Figure 9:**
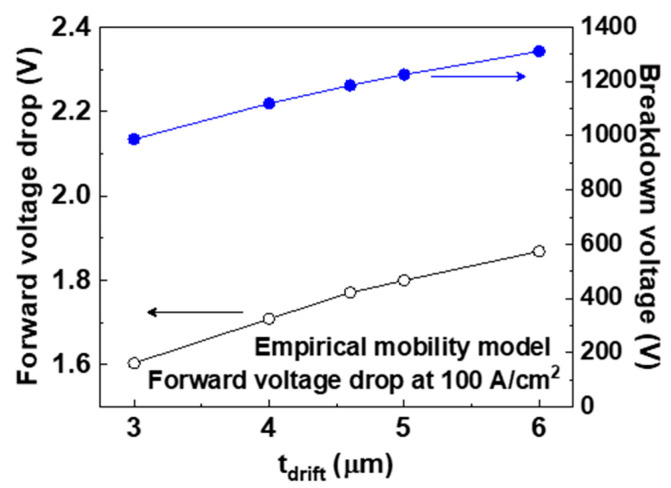
Simulated forward voltage drop at 100 A/cm^2^ (*V_f_*) and breakdown voltage of the p-type diamond pseudo-vertical Schottky barrier diodes (SBDs) with values of *t_drift_* of 3.0, 4.0, 4.6, 5.0, and 6.0 μm. The empirical mobility model used for the forward current–voltage (I–V) characteristics at 300 K.

Figure 9 shows the simulated *V_f_* and breakdown voltage of the p-type pseudo-vertical diamond SBDs with values of *t_drift_* of 3.0, 4.0, 4.6, 5.0, and 6.0 μm. We used the empirical mobility model for the forward I–V. The forward I–V characteristics are also included in the Supplementary Material as Figure S4. The values of *V_f_* with *t_drift_* of 3.0, 4.0, 4.6, 5.0, and 6.0 μm were 1.60, 1.71, 1.77, 1.80, and 1.87 at 100 A/cm^2^, respectively. Converting these values to *R_on,sp_* gives 5.1, 6.2, 6.8, 7.1, and 7.8 mΩ∙cm^2^, respectively. The breakdown voltage of the devices with values of *t_drift_* of 3.0, 4.0, 4.6, 5.0, and 6.0 μm were 990, 1120, 1190, 1230, and 1310 V, respectively. An increase in *t_drift_* increased the resistance of the p- drift layer, which in turn decreased the forward current. However, this expanded the depletion region at the p- drift layer, which increased the breakdown voltage.

**Figure 10:**
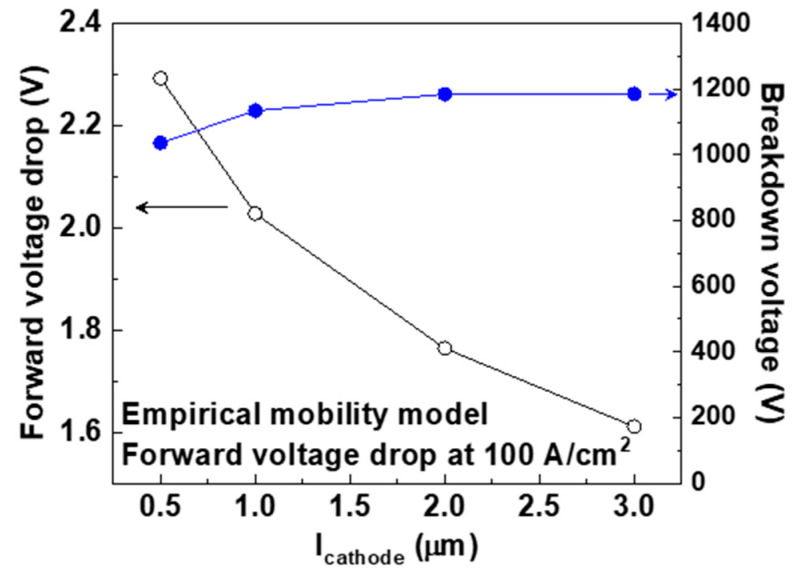
Simulated forward voltage drop at 100 A/cm^2^ (*V_f_*) and breakdown voltage of the p-type diamond pseudo-vertical Schottky barrier diodes (SBDs) with values of *l_cathode_* of 0.5, 1.0, 2.0, and 3.0 μm. The empirical mobility model was used at 300 K.

Figure 10 shows the simulated *V_f_* and breakdown voltage of the devices with values of *l_cathode_* of 0.5, 1.0, 2.0, and 3.0 μm, respectively. The empirical mobility model used for the forward I–V characteristics. The forward I–V characteristics are also included in the Supplementary Material as Figure S5. The values of *V_f_* of the devices with *l_cathode_* of 0.5, 1.0, 2.0, and 3.0 were 2.29, 2.03, 1.77, and 1.61 V at 100 A/cm^2^, respectively. Converting these values to *R_on,sp_* gives 12.0, 9.4, 6.8, and 5.2 mΩ∙cm^2^, respectively. The narrow Schottky contact increased the current crowding and on-resistance during conduction. When the *l_cathode_* decreased from 1.0 to 0.5 μm, the breakdown voltage decreased slightly from 1140 to 1040 V. This was caused by a decrease in the depletion area under the Schottky contact. When the *l_cathode_* was longer than 1.0 μm, the breakdown voltage did not change considerably.

Finally, we simulated the forward and reverse characteristics of the p-type pseudo-vertical diamond SBDs with empirical mobility and modified impact ionization models [25,29]. The empirical mobility model [29] was suitable for the numerical simulation at the low field because it is based on experimental results. The device achieved a low value of *R_on,sp_* of 6.8 mΩ∙cm^2^, a high breakdown voltage of 1190 V, and a high figure-of-merit of 210 MW/cm^2^. The figure-of-merit was the square of the breakdown voltage divided by the value of *R_on,sp_*. The diamond devices promise low power loss and high energy conversion efficiency in the power systems.

## 4. Conclusions

The forward I–V characteristics of p-type pseudo-vertical diamond SBDs were investigated; the use of various mobility models was important because this resulted in different values of *V_f_* and *R_on,sp_*. The analytic mobility model uses a mobility that changes with doping concentration, while the constant mobility model fixes the mobility absolutely. The Lombardi CVT mobility model considers the perpendicular electric field, doping concentration, and temperature-dependent scattering. This led to mobility degradation at the edge of the Schottky contact where the electric field was concentrated. The empirical mobility model had the advantage of being based on experimental results. The p-type diamond pseudo-vertical SBD achieved high performance, with a value of *R_on,sp_* of 6.8 mΩ∙cm^2^, a breakdown voltage of 1190 V, and a figure-of-merit of 210 MW/cm^2^. Diamond power devices with a high breakdown field and high thermal conductivity are suitable for power switching devices that could replace Si and SiC devices.

## Figures and Tables

**Table 1:** Hole mobility, forward voltage drop (*V_f_*), and specific on-resistance (*R_on,sp_*) of the p-type diamond pseudo-vertical Schottky barrier diode (SBD) simulated using the constant mobility model with *μ_const_* of 1000, 2000, 3000, and 4000 cm^2^/Vs. The lattice temperature was 300 K.

μ_const_ (cm^2^/Vs)	Hole Mobility (cm^2^/Vs)	Forward Voltage Drop (V)	Specific on-Resistance (mΩ∙cm^2^)
1000	1000	2.47	14.1
2000	2000	1.76	7.0
3000	3000	1.52	4.6
4000	4000	1.40	3.4

**Table 2:** Built-in potential, hole mobility, forward voltage drop (*V_f_*) and specific on-resistance (*R_on,sp_*) of the p-type diamond pseudo-vertical Schottky barrier diode (SBD) simulated using the constant, analytic, Lombardi CVT, and empirical mobility models. The constant mobility model used the *μ_const_* of 2000 cm^2^/Vs.

Mobility Model	Temperature (K)	Built-in Potential (V)	Hole Mobility (cm^2^/Vs)	Forward Voltage Drop (V)	Specific on-Resistance (mΩ∙cm^2^)
Constant	200	1.2	3670	1.57	3.8
	300	1.1	2000	1.76	7.0
	400	1.0	1300	2.01	10.9
	500	0.8	930	2.30	14.8
Analytic	200	1.2	720	3.16	19.3
	300	1.1	790	2.86	17.5
	400	1.0	800	2.69	17.4
	500	0.8	790	2.57	17.5
Lombardi CVT	200	1.2	-	2.84	17.2
	300	1.1	-	5.17	40.8
	400	1.0	-	9.11	82.0
	500	0.8	-	15.49	145.8
Empirical	300	1.1	1990	1.77	6.8
	400	1.0	820	2.65	16.8
	500	0.8	410	4.20	33.7
Experimental [15]	300	2.1	-	-	18.5

## Supplementary Materials

The following are available online at https://www.mdpi.com/2072-666X/11/6/598/s1, Figure S1: Forward current–voltage (I–V) characteristics of the p-type diamond pseudo-vertical Schottky barrier diode (SBD) simulated using the analytic mobility model at 200, 300, 400, and 500 K, Figure S2: Two-dimensional electric field of the p-type diamond pseudo-vertical Schottky barrier diode (SBD) simulated using the Lombardi CVT mobility model, Figure S3: Forward current–voltage (I–V) characteristics of the p-type diamond pseudo-vertical Schottky barrier diode (SBD) simulated using the Lombardi CVT mobility model at 200, 300, 400, and 500 K, Figure S4: Forward current–voltage (I–V) characteristics of the p-type diamond pseudo-vertical Schottky barrier diodes (SBDs) simulated using the empirical mobility model with *t_drift_* of 3.0, 4.0, 4.6, 5.0, and 6.0 μm. Figure S5: Forward current–voltage (I–V) characteristics of the p-type diamond pseudo-vertical Schottky barrier diodes (SBDs) simulated using the empirical mobility model with *l_drift_* of 0.5, 1.0, 2.0, and 3.0 μm.

## References

[1] Isberg J., Hammersberg J., Johansson E., Wikström T., Twitchen D.J., Whitehead A.J., Coe S.E., Scarsbrook G.A. (2002). High Carrier Mobility in Single-Crystal Plasma-Deposited Diamond. Science.

[2] Chicot G., Eon D., Rouger N. (2016). Optimal drift region for diamond power devices. Diam. Relat. Mater..

[3] Kasu M. (2016). Diamond field-effect transistors for RF power electronics: Novel NO_2_ hole doping and low-temperature deposited Al_2_O_3_ passivation. Jpn. J. Appl. Phys..

[4] Hitchcock C., Chow T.P. (2020). Degradation of forward current density with increasing blocking voltage in diamond Schottky-pn diodes. Diam. Relat. Mater..

[5] Zhao D., Liu Z., Wang J., Yi W., Wang R., Wang K., Wang H. (2019). Performance Improved Vertical Diamond Schottky Barrier Diode with Fluorination-Termination Structure. IEEE Electron Device Lett..

[6] Matsumoto T., Kato H., Oyama K., Makino T., Ogura M., Takeuchi D., Inokuma T., Tokuda N., Yamasaki S. (2016). Inversion channel diamond metal-oxide-semiconductor with normally-off characteristics. Sci. Rep..

[7] Liu J., Teraji T., Da B., Ohsato H., Koide Y. (2020). Effect of Annealing Temperature on Performances of Boron-Doped Diamond Metal–Semiconductor Field-Effect Transistors. IEEE Trans. Electron Devices.

[8] Ren Z., Lv D., Xu J., Zhang J., Zhang J., Su K., Zhang C., Hao Y. (2020). High temperature (300 °C) ALD grown Al_2_O_3_ on hydrogen terminated diamond: Band offset and electrical properties of the MOSFETs. Appl. Phys. Lett..

[9] Hicks M.-L., Pakpour-Tabrizi A.C., Jackman R.B. (2019). Polishing, preparation and patterning of diamond for device applications. Diam. Relat. Mater..

[10] Wang W., Wang Y.-F., Zhang M., Wang R., Chen G., Chang X., Lin F., Wen F., Jia K., Wang H.-X. (2020). An Enhancement-Mode Hydrogen-Terminated Diamond Field-Effect Transistor with Lanthanum Hexaboride Gate Material. IEEE Electron Device Lett..

[11] Sun C., Hao T., Li J., Ye H., Gu C.-Z. (2020). The design and performance of hydrogen-terminated diamond metal-oxide-semiconductor field-effect transistors with high k oxide HfO_2_. Micro Nano Eng..

[12] Koizumi S., Umezawa H., Pernot J., Suzuki M. (2018). Power Electronics Device Applications of Diamond Semiconductors.

[13] Thonke K. (2003). The boron acceptor in diamond. Semicond. Sci. Technol..

[14] Shikata S. (2016). Single crystal diamond wafers for high power electronics. Diam. Relat. Mater..

[15] Ikeda K., Umezawa H., Tatsumi N., Ramanujam K., Shikata S.-I. (2009). Fabrication of a field plate structure for diamond Schottky barrier diodes. Diam. Relat. Mater..

[16] Kumaresan R., Umezawa H., Tatsumi N., Ikeda K., Shikata S. (2009). Device processing, fabrication and analysis of diamond pseudo-vertical Schottky barrier diodes with low leak current and high blocking voltage. Diam. Relat. Mater..

[17] Umezawa H., Tatsumi N., Kato Y., Shikata S.-I. (2013). Leakage current analysis of diamond Schottky barrier diodes by defect imaging. Diam. Relat. Mater..

[18] Dutta M., Koeck F., Li W., Nemanich R.J., Chowdhury S. (2017). High Voltage Diodes in Diamond using (100)- and (111)- Substrates. IEEE Electron Device Lett..

[19] Ding H., Isoird K., Schneider H., Koné S., Civrac G. (2010). Basic parameters and models in simulation of CVD diamond devices. Diam. Relat. Mater..

[20] Thion F., Isoird K., Planson D., Locatelli M.-L., Ding H. (2011). Simulation and design of junction termination structures for diamond Schottky diodes. Diam. Relat. Mater..

[21] Arbess H., Isoird K., Zerarka M., Schneider H., Locatelli M.-L., Planson D. (2015). High termination efficiency using polyimide trench for high voltage diamond Schottky diode. Diam. Relat. Mater..

[22] Maréchal A., Rouger N., Crébier J.-C., Pernot J., Koizumi S., Teraji T., Gheeraert E. (2014). Model implementation towards the predicition of J(V) characteristics in diamond bipolar device simulations. Diam. Relat. Mater..

[23] Hathwar R., Dutta M., Koeck F., Nemanich R.J., Chowdhury S., Goodnick S.M. (2016). Temperature dependent simulation of diamond depleted Schottky PIN diodes. J. Appl. Phys..

[24] Chou J.-P., Retzker A., Gali A. (2017). Nitrogen-Terminated Diamond (111) Surface for Room-Temperature Quantum Sensing and Simulation. Nano Lett..

[25] Kang D.-W., Chang H.N., Ha M.-W. (2017). Numerical simulation of p-type diamond Schottky barrier diodes for high breakdown voltage. Jpn. J. Appl. Phys..

[26] Caughey D., Thomas R. (1967). Carrier mobilities in silicon empirically related to doping and field. Proc. IEEE.

[27] Shaw J.G., Hack M. (1988). An analytic model for calculating trapped charge in amorphous silicon. J. Appl. Phys..

[28] Lombardi C., Manzini S., Saporito A., Vanzi M. (1988). A physically based mobility model for numerical simulation of nonplanar devices. IEEE Trans. Comput. Des. Integr. Circuits Syst..

[29] Volpe P.-N., Pernot J., Muret P., Omnès F. (2009). High hole mobility in boron doped diamond for power device applications. Appl. Phys. Lett..

[30] Silvaco International (2009). Atlas User’s Manual.

[31] Miyata K., Nishimura K., Kobashi K. (1995). Device simulation of submicrometer gate p^+^-i-p^+^ diamond transistors. IEEE Trans. Electron Devices.

[32] Pernot J., Volpe P.N., Omnès F., Muret P., Mortet V., Haenen K., Teraji T. (2010). Hall hole mobility in boron-doped homoepitaxial diamond. Phys. Rev. B.

[33] Rashid S.J., Tajani A., Twitchen D.J., Coulbeck L., Udrea F., Butler T., Rupesinghe N.L., Brezeanu M., Isberg J., Garraway A. (2008). Numerical Parameterization of Chemical-Vapor-Deposited (CVD) Single-Crystal Diamond for Device Simulation and Analysis. IEEE Trans. Electron Devices.

[34] Selberherr S. (1984). Analysis and Simulation of Semiconductor Devices.

[35] Van Overstraeten R., De Man H. (1970). Measurement of the ionization rates in diffused silicon p-n junctions. Solid State Electron..

[36] Chynoweth A.G. (1958). Ionization Rates for Electrons and Holes in Silicon. Phys. Rev..

