# Supplementary material for: Mobility Models Based on Forward Current-Voltage Characteristics of P-type Pseudo-Vertical Diamond Schottky Barrier Diodes
Source: Micromachines (Basel). 2020 Jun 18;11(6):598. doi: 10.3390/mi11060598 (PMC7344954; doi:10.3390/mi11060598)
Supplement: Supplementary file 1 [file micromachines-11-00598-s001.pdf]

micromachines

Mobility Models Based on Forward Current–voltage Characteristics of P-type Pseudo-vertical Diamond Schottky Barrier Diodes

Min-Woo Ha [1], Ogyun Seok [2], Hojun Lee [1], and Hyun Ho Lee [3,*]

[1] Department of Electrical Engineering, Myongji University, 116 Myongji-ro, Cheoin-gu, Yongin, Gyeonggi 17058, Korea; isobar@mju.ac.kr (M.-W.H.); 80180234@mju.ac.kr (H.L.)
[2] Korea Electrotechnology Research Institute, Changwon, Gyeongnam 51543, Korea; ogseok@keri.re.kr (O.S.)
[3] Department of Chemical Engineering, Myongji University, 116 Myongji-ro, Cheoin-gu, Yongin, Gyeonggi 17058, Korea
* Correspondence: hyunho@mju.ac.kr; Tel.: +82-31-330-6392

Supplementary Material

Figure S1 shows the forward current–voltage (I–V) characteristics of the p-type pseudo-vertical Schottky barrier diode (SBD) simulated using the analytic mobility model at 200, 300, 400, and 500 K. Figure S2 exhibits the two-dimensional electric field of the p-type diamond pseudo-vertical SBD simulated using the Lombard CVT mobility model. The anode voltage (V_a) and lattice temperature were 2 V and 300 K. Figure S3 is the forward I–V characteristics of the p-type diamond pseudo-vertical SBD simulated using the Lombardi CVT model at 200, 300, 400, and 500 K.

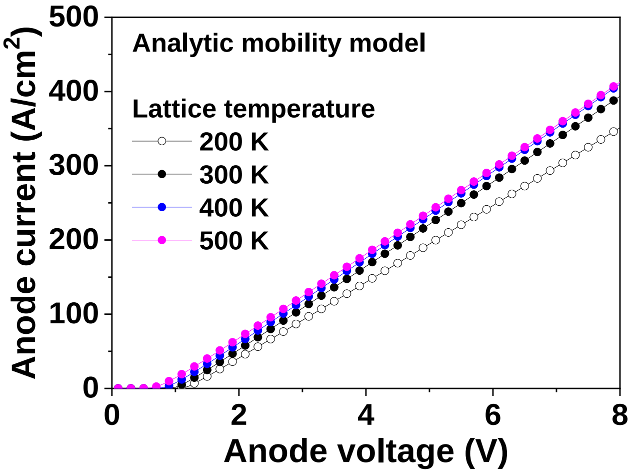

Figure S1. Forward current–voltage (I–V) characteristics of the p-type diamond pseudo-vertical Schottky barrier diode (SBD) simulated using the analytic mobility model at 200, 300, 400, and 500 K. The metal work function and hole saturation velocity were 5.65 eV and 2.7 × 10^7 cm/s respectively.

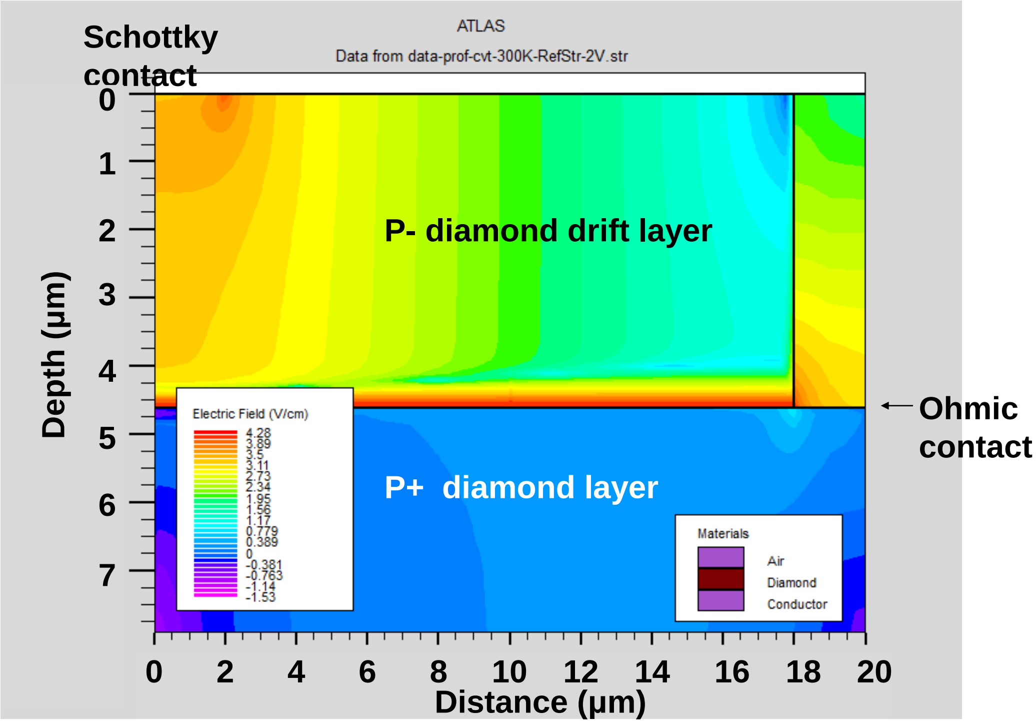

Figure S2. Two-dimensional electric field of the p-type diamond pseudo-vertical Schottky barrier diode (SBD) simulated using the Lombardi CVT mobility model. The metal work function, hole saturation velocity, lattice temperature, and anode voltage (V_a) were 5.65 eV, 2.7 × 10^7 cm/s, 300 K, and 2 V, respectively.

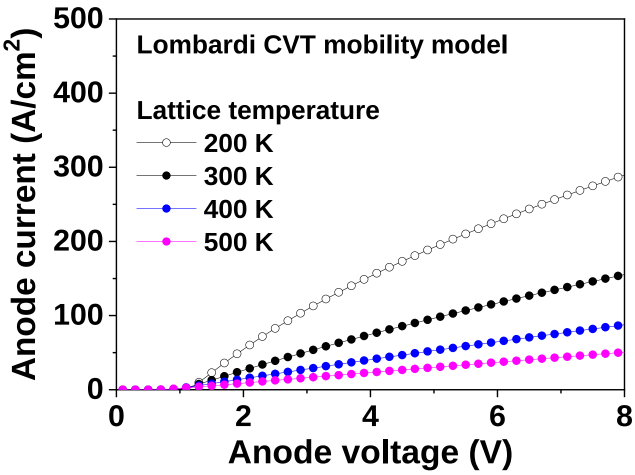

Figure S3. Forward current–voltage (I–V) characteristics of the p-type diamond pseudo-vertical Schottky barrier diode (SBD) simulated using the Lombardi CVT mobility model at 200, 300, 400, and 500 K. The metal work function and hole saturation velocity were 5.65 eV and 2.7 × 10^7 cm/s, respectively.

Figure S4 is the forward I–V characteristics of the p-type diamond pseudo-vertical SBDs simulated using the empirical mobility model at 300 K. The thickness of the P- drift layer (t_{drift}) were 3.0, 4.0, 4.6, 5.0, and 6.0 μm. Figure S5 shows the forward I–V characteristics of the p-type diamond pseudo-vertical SBDs simulated using the empirical mobility model at 300 K. The length of the cathode ($l_{cathode}$) were 0.5, 1.0, 2.0, and 3.0 μm.

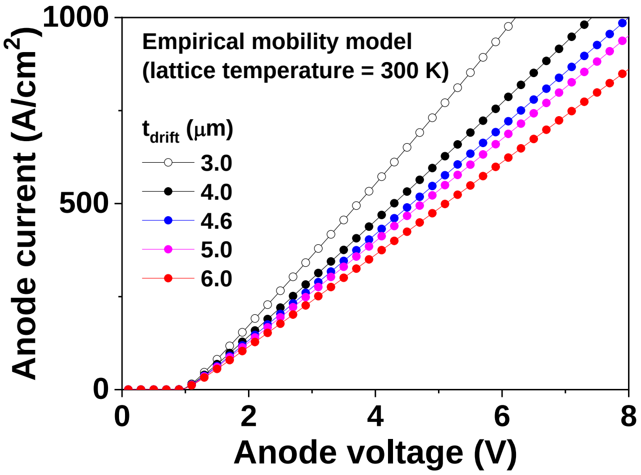

Figure S4. Forward current–voltage (I–V) characteristics of the p-type diamond pseudo-vertical Schottky barrier diodes (SBDs) simulated using the empirical mobility model at 300 K. The values of t_{drift} were 3.0, 4.0, 4.6, 5.0, and 6.0 μm, respectively. The metal work function, hole saturation velocity, hole mobility, and β were 5.65 eV, 2.7 × 10^7 cm/s, 1,990 cm^2/Vs, and 3.092, respectively.

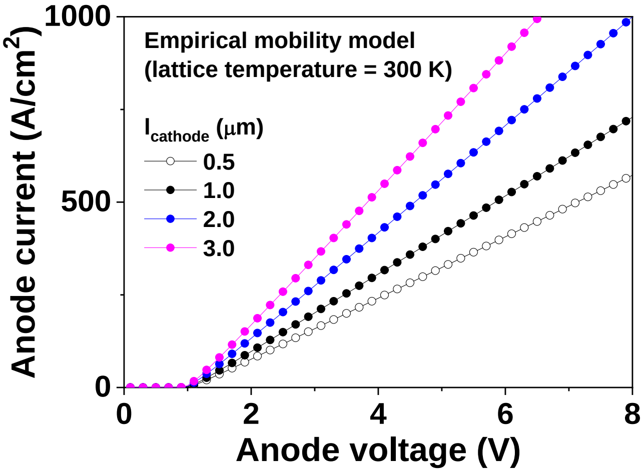

Figure S5. Forward current–voltage (I–V) characteristics of the p-type diamond pseudo-vertical Schottky barrier diodes (SBDs) simulated using the empirical mobility model at 300 K. The values of $l_{cathode}$ were 0.5, 1.0, 2.0, and 3.0 μm. The metal work function, hole saturation velocity, hole mobility, and β were 5.65 eV, 2.7 × 10^7 cm/s, 1,990 cm^2/Vs, and 3.092, respectively.

© 2020 by the authors. Submitted for possible open access publication under the terms and conditions of the Creative Commons Attribution (CC BY) license (http://creativecommons.org/licenses/by/4.0/).
